# 3-Methoxy-Phencyclidine Induced Psychotic Disorder: A Literature Review and an ^18^F-FDG PET/CT Case Report

**DOI:** 10.3390/ph17040452

**Published:** 2024-03-31

**Authors:** Maria Pepe, Marco Di Nicola, Fabrizio Cocciolillo, Stefania Chiappini, Giovanni Martinotti, Maria Lucia Calcagni, Gabriele Sani

**Affiliations:** 1 Department of Neuroscience, Section of Psychiatry, Università Cattolica del Sacro Cuore, L.go F. Vito 1, 00168 Rome, Italy; 2 Department of Psychiatry, Fondazione Policlinico Universitario A. Gemelli IRCCS, L.go A. Gemelli 8, 00168 Rome, Italy; 3 Nuclear Medicine Unit, Department of Diagnostic Imaging, Oncological Radiotherapy and Hematology, Fondazione Policlinico Universitario A. Gemelli IRCCS, L.go A. Gemelli 8, 00168 Rome, Italy; 4 Department of Neurosciences, Imaging and Clinical Sciences, Università degli Studi G. D’Annunzio, Chieti-Pescara, Via dei Vestini 31, 66013 Chieti, Italy; 5 School of Medical Sciences, UniCamillus International University of Medical Sciences, Via di S. Alessandro 8, 00131 Rome, Italy; 6 Psychopharmacology, Drug Misuse and Novel Psychoactive Substances Research Unit, School of Life and Medical Sciences, University of Hertfordshire, Hatfield AL10 9EU, UK; 7 Institute of Nuclear Medicine, Università Cattolica del Sacro Cuore, L.go F. Vito 1, 00168 Rome, Italy

**Keywords:** NPS, 3-MeO-PCP, substance-induced psychosis, cognitive functioning, neuroimaging

## Abstract

New Psychoactive Substances (NPS) are modifying the drug scenario worldwide and have become a public health concern because of their toxicological profiles and their harmful physical/psychological effects. 3-Methoxy-Phencyclidine (3-MeO-PCP), a non-competitive antagonist of glutamate N-methyl-D-aspartate (NMDA) receptors, belongs to the phencyclidine-like subfamily of arylcyclohexylamines and has gained attention for its toxic, sometimes fatal, effects. Despite several cases of intoxication and death reported in the literature, little is known about substance-induced psychotic disorders (SIP) and potential cognitive impairment following 3-MeO-PCP intake. This literature review aimed to summarize available evidence about 3-MeO-PCP mechanisms of action and physical and psychotropic effects and to spread preliminary findings about persistent psychotic symptoms and impaired cognitive functioning. Additionally, the case of an SIP is reported in a 29-year-old man with small oral intakes of 3-MeO-PCP over two weeks until a high dose ingestion. Psychometric and neuropsychological assessment and brain [^18^F]-fluorodeoxyglucose positron emission tomography integrated with computed tomography were used to support clinical description. Identifying and addressing the characteristic clinical features and neural substrates of NPS-induced psychoses might help clinicians with a more precise differentiation from other psychotic disorders. Although further studies are required, phenotyping the cognitive profile of NPS users might provide targets for tailored therapeutic approaches.

## 1. Introduction

### 1.1. The Emerging Phenomenon of NPS

‘New Psychoactive Substances’ (NPS) is an umbrella term that was officially used by the United Nations Office on Drugs and Crime (UNODC) to refer to ‘substances of abuse, either in a pure form or a preparation, that are not controlled by the 1961 Single Convention on Narcotic Drugs or the 1971 Convention on Psychotropic Substances, but which may pose a health threat’ [1]. Such unregulated psychoactive compounds have become a public health concern since their spread from the 2000s onwards [2] and peaked in 2021 after several years of stabilization, with a cumulative number of 1184 substances currently identified worldwide [3].

The COVID-19 pandemic also influenced the illicit drug market, and more than 200 cases of NPS consumption, including both intoxications and fatalities, have been reported from different countries [4]. Indeed, the European Monitoring Centre for Drugs and Drug Addiction (EMCDDA) was tracking around 930 new substances at the end of 2022 and reported alarming estimates of lifetime and last-year use among adolescents and young adults [5]. Accordingly, Italian data showed an increasing trend for NPS use in the general population in 2022 and pointed to these compounds as the second most-used illegal substance class, after cannabis, among students aged 15–19 years, with up to 6% reporting at least one intake in the last 12 months [6].

Being synthetic derivates of traditional drugs with minor functional or chemical changes, NPS display different pharmacological properties and are generally excluded from the list of registered narcotic products, having the possibility to be sold as legal alternatives [2,7]. Also known as “designer drugs” or “bath salts”, NPS have been promoted as “legal highs” and are rapidly modifying the drug scenario worldwide [5] because of their relatively low cost, ready availability through online purchasing, and undetectability at routine drug screening tests [8,9].

Over the last few years, this emerging phenomenon has been monitored by the authorities, and many activities have been implemented to identify and schedule NPS as law-controlled substances [10]. However, although information is regularly spread by official reports and alerts, the increasing number of available NPS and the wide structural variety make it difficult to understand their toxicological profiles and the potentially harmful physical, psychological, and social effects [8,9].

### 1.2. About 3-Methoxy-Phencyclidine

NPS include different chemical classes like synthetic cathinone and cannabinoids, piperazines, phenethylamines, tryptamines, and phencyclidines [11]. Analogs and substitutes of 1-1-phenylcyclohexyl-piperidine (PCP), a dissociative anesthetic, constitute the phencyclidine class and have spread as street drugs since the 1960–1970s [12], following PCP removal from the market because of its psychotic-like effects [8]. PCP, also known as “angel dust”, and related compounds have been shown to exert their psychotropic effects via the antagonism of glutamate *N*-methyl-D-aspartate (NMDA) receptors, a mechanism involved in both schizophrenia-like positive and negative symptoms, as well as in cognitive alterations [13]. Since then, PCP-treated animals served as preclinical research models of schizophrenia, supporting the role of glutamatergic dysfunction in this disease [14].

Arylcyclohexylamines (ACHs) are PCP derivatives with three distinct subcategories obtained through modifications of the aryl ring, and although a recreational interest has been reported only for a few of these compounds [15], the PCP-like subfamily has gained increasing attention for its extremely toxic, sometimes fatal, effects [12]. After the peak in PCP consumption ten years ago, its derivatives and other novel dissociatives have progressively expanded on the market, being increasingly reported by drug users, especially adolescents and young adults, and being detected in clinical drug analyses more often than PCP itself and more frequently than in the pre-pandemic period [16,17,18,19].

1-[1-(3-methoxyphenyl)cyclohexyl]piperidine (i.e., 3-MeO-PCP) was the last of the isomers synthesized in 1979 [20] and has been placed under regulatory control in many countries since the first EMCDDA notification in 2012 because of its risks and misuse potential [21,22]. Although the consumption of 3-MeO-PCP remained relatively stable in the very last years compared to the increase in past-year and past-month use of other substances (e.g., cannabis, ketamine, synthetic cathinone, novel psychedelics), it has been identified in most cases of single-substance intoxication of PCP analogs [3,6,16,22].

#### 1.2.1. General Pharmacology

The non-competitive antagonism of glutamate NMDA receptors is the primary mechanism of action of 3-MeO-PCP. Additionally, this compound showed minor effects on the serotonin transporter [23] and sigma-1 receptors, along with a functional interaction with the dopamine transporter [24]. Like other dissociative hallucinogens and PCP derivatives, the NMDA antagonism is responsible for the psychotropic effects, and 3-MeO-PCP showed a greater binding affinity than PCP [9,12].

3-MeO-PCP is usually consumed through sniffing, followed by oral intake. However, further routes of administration are available (i.e., smoking, injection), with differences in the action onset and duration [22,25]. 3-MeO-PCP-induced effects arise between 30 and 90 min after ingestion, may last from 4 to 48 h, and can follow the consumption of doses as low as 1 mg [22]. 3-MeO-PCP undergoes extensive metabolism, with several phase I and II metabolites produced through hydroxylation, carboxylation, and O-demethylation processes [26].

A thorough measurement of blood concentrations leading to toxicity and fatality is missing due to the lack of documented intoxication cases and information about the preservation status of samples. Indeed, the stability of 3-MeO-PCP and its metabolites in post-mortem specimens is unclear, and the frequent co-presence of other substances prevents from clearly identifying the lethal dose [25]. Nevertheless, nasal/oral consumption of more than 12/15 milligrams could be considered a heavy dose [22]. Blood ranges of 50–3525 ng/mL were measured in fatal intoxications, with a concentration of approximately 380 ng/mL identified in one case where death could certainly be ascribed to 3-MeO-PCP [25,27].

#### 1.2.2. Intoxication and Clinical Manifestations

Cardiovascular, respiratory, and cerebral alterations, including hypertension, tachycardia, pulmonary edema, and brain swelling, have been reported among the acute pathophysiological consequences of 3-MeO-PCP intake and require symptomatic treatments (e.g., hyperhydration, tracheal intubation) and constant monitoring of vital signs and biochemical parameters [9,12]. Recent observations suggested a potential exacerbating effect of physical effort on the toxicity of this compound, highlighting a higher risk associated with the use of 3-MeO-PCP in recreational contexts or any situation that may be associated with increased heart rate, blood pressure, and body temperature [25].

Although data on the toxicity profile of chronic consumption are still inconsistent, bladder and liver damage, digestive disorders with significant abdominal pain, and neurological symptoms (including memory and attention deficits, blunted reactivity, impaired perception of time, and color) may characterize the prolonged use of 3-MeO-PCP [12,28]. As for the psychoactive consequences, 3-MeO-PCP users generally report euphoria, increased empathy, dissociation, and hallucinations as the immediate, desired effects, although behavioral alterations with psychomotor agitation, confusion, cognitive impairment, and severe psychotic symptoms can occur [8].

To date, several 3-MeO-PCP intoxication cases, both fatal and nonfatal, have been described in the literature [3,8,25,26,27,29,30,31,32,33,34,35,36,37,38,39,40,41,42,43,44,45,46,47,48]. It is noteworthy that 3-MeO-PCP figured as the only substance assumed in less than one-third of reports, eight nonfatal and one fatal, and that the concurrent intake of multiple substances can modify the clinical picture and contribute to a more severe presentation [25,29]. Available reports with publication details, numbers, and brief descriptions of cases (i.e., gender, age, toxicological profile, and outcome) are summarized in Table 1.

Signs and symptoms of nonfatal intoxication include erratic behavior, confusion, disorientation, dissociation, hallucinations, and other signs of psychosis, often associated with altered neurological status and amnesia regarding the intoxication timeframe [27,30,31]. Psychomotor agitation was particularly prominent in subjects with multiple substance abuses [29]. Four cases of patients hospitalized after 3-MeO-PCP ingestion have been described by Italian reports that described, among other symptoms, neurological impairment and delirium with agitation and euphoria still detectable after 24 h hospital monitoring [8,32].

#### 1.2.3. The Effects of Other PCP-Derivatives and Novel Dissociative Compounds

The physical and psychopathological effects of the consumption of other PCP derivatives and novel dissociative compounds only partially differ from those induced by 3-MeO-PCP, making it difficult for clinicians to differentiate clinical pictures. Estimates of PCP use and related emergency department referrals were alarmingly widespread in 2013 [3]. While subjects reported distorted perceptions of sight and sound, dissociation from the environment, and out-of-body experiences as the desired effects of the compound, the acute psychopathological symptoms included memory impairments, altered perception of time, slowness, anxiety, apathy, irritability, psychotic symptoms, violent behaviors, and states of stupor until coma. As for the physical effects, increased breathing rate, elevated blood pressure, tachycardia, flushing and excessive sweating, nausea and vomiting, blurred vision, loss of balance, dizziness, kidney failure, seizures, cardiac arrest, and cerebrovascular accidents have been described [49].

Among PCP first-generation isomers synthesized between the 1960s and 1990s, 4-MeO-PCP and 3-OH-PCP started spreading on the narcotics market in 2008 and 2009, respectively. Despite the almost overlapping effects, 4-MeO-PCP proved to be less potent than PCP and 3-MeO-PCP, with few intoxications reported so far, while no cases have been described for 3-OH-PCP, although it is active at low doses [3].

As for the effects of other dissociatives, depersonalization and dissociation with intense detachment, near-death experiences, and auditory and visual hallucinations are generally sought by ketamine users. However, acute symptoms also include anxiety, flashbacks, and a set of physical effects (i.e., tachycardia, agitation, hypertension, nausea, slurred speech, dizziness, and collapse) that can result in accidental injury, drowning, or death from hypothermia. Finally, methoxetamine should be mentioned for its desired effects, such as relaxation, euphoria, and more intense, longer-lasting sympathomimetic activity than ketamine, which might combine with physical symptoms like blackouts, cerebellar toxicity, and cardiac arrhythmias [50].

Cognitive impairment and mood shifts/alterations have been described after chronic use of PCP and methoxetamine, while perceptual disorders seem to be more common after ketamine and methoxetamine intake. The development of addiction with withdrawal signs and symptoms has been discussed for both PCP/PCP-analogues and ketamine, while more specific chronic effects may include anxiety disorders and suicidal thoughts for PCP/PCP-analogues and impairment of attention and recall, psychosis, and hallucinogen-persisting perception disorder for ketamine [50].

### 1.3. Limitations and ‘Unmet Needs’

The psychoactive consequences of 3-MeO-PCP, both in the short and long term, have only been partially understood, possibly because of some limitations of the available evidence. First, most reports from the literature have described fatal intoxications with post-mortem detection of 3-MeO-PCP. Other studies focused on the acute presentation after admission to the emergency department or during the intensive care unit stay, where patients are mostly intubated and unconscious. Further, because of the pharmacodynamic/kinetic interactions between compounds, the concurrent intake of several substances (primarily alcohol and stimulants like amphetamines and cocaine, followed by sedative-hypnotics/anxiolytics) may modify the clinical presentation, increase severity, and hinder the exact attribution of the effects to one substance over another. Lastly, once the acute symptoms of intoxication are remitted, patients are usually discharged from the hospital within a few days, and information on what happens afterward is missing [25].

Illicit substances can induce transient psychotic symptoms during an acute phase but also lead to a more persistent syndrome with predominant delusions and/or hallucinations occurring during or immediately after intoxication/withdrawal, which has been officially recognized by the Diagnostic and Statistical Manual of Mental Disorder (DSM) as a substance-induced psychotic disorder [51,52]. Although substance-induced psychoses (SIP) have become a common issue in clinical practice and some evidence has been collected about the psychopathological consequences of NPS [49,50], the description of cases of substance-induced psychotic disorders following 3-MeO-PCP consumption and its potential effects on brain functions is still lacking. To our knowledge, there is only one report in the literature about the consequences of NPS from a neuroimaging perspective, which described the effects on brain metabolism and activity of an NMDA receptor antagonist belonging to the same ACH class as 3-MeO-PCP [53].

Hereafter, we present the case of a patient with oral ingestion of 3-MeO-PCP who experienced prolonged psychotic symptoms following the intake and displayed impaired brain functioning at neuropsychological and neuroimaging investigations.

## 2. Case History

A 29-year-old man referred to the “Centro Psichiatrico Integrato per la ricerca, la prevenzione e la cura delle Dipendenze” (CePID), Department of Psychiatry of Fondazione Policlinico Universitario Agostino Gemelli IRCCS in Rome, in an outpatient setting, for psychotic symptoms and behavioral alterations. The medical history outlined normal child development and the absence of relevant organic diseases, except for the presence of genetic variants of coagulation factors and enzymes (heterozygous mutation of Leiden factor V, homozygous C677T mutation of MTHFR). While there was no history of family psychiatric disorders, the onset of psychiatric symptoms occurred in adolescence, around the age of 17, with depressed mood, social anxiety, ruminative thoughts about social functioning and sexual orientation, and subsequent use of alcohol and psychotropic substances with a self-medicating purpose. The patient underwent a first psychiatric consultation during the last years of high school and was prescribed different psychopharmacological therapies (i.e., antidepressants, mood stabilizers, anxiolytics/sedative-hypnotics, variously combined) but displayed discontinuous treatment adherence and poor symptomatic improvement. As a young adult, he declares the intake of multiple substances without meeting the criteria for a specific Substance Use Disorder (SUD), as well as the misuse of prescription and over-the-counter drugs (including dextromethorphan, quetiapine, selegiline, and tramadol). The patient underwent a detoxification program and, after four weeks of abstinence, was prescribed psychopharmacotherapy targeting anxious-depressive symptoms (paroxetine 20 mg/day, quetiapine extended-release 150 mg/day, valproate 750 mg/day, and trazodone extended release 75 mg/day). However, over the past few weeks before the following evaluation, he developed a manic-like episode with psychotic features, including both visual and tactile hallucinations, paranoid delusions, severe dissociation, and insomnia, as referred to by his relatives and still detectable at the clinical interview. The patient reports having started the online purchase of NPS a month before and, specifically, to have consumed small dosages of 3-MeO-PCP for at least two weeks until the oral ingestion of an unspecified but large amount (almost 300–500 mg referred) about three days before the evaluation. The consequences of this massive intake were significant despair, a sense of impending doom, psychomotor agitation, and aggressive behaviors towards the family and himself, followed by total amnesia. Relatives reported that the presence of 3-MeO-PCP in blood samples (concentration not quantified) was confirmed through gas and liquid chromatography coupled to mass spectrometry techniques during a previous attendance to the emergency department for a nasal fracture that occurred during an episode of psychomotor agitation.

### 2.1. Methods

A clinical interview and a psychometric assessment were carried out at baseline and after one week via the following clinician-rated scales and patient self-reported questionnaires: *Brief Psychiatric Rating Scale* (BPRS) [54], *Clinical Global Impression, Severity, and Improvement* (CGI-S/I) [55], and *Self-report Symptom Checklist Inventory 90—Revised Version* (SCL90-R) [56]. A neuropsychological evaluation was performed through the pen-and-paper versions of the *Digit Symbol Substitution Test* (DSST) [57] and the *Trail Making Test* (TMT) [58], administered according to the guidelines outlined by Strauss et al. [59] and scored respectively through the number of correct symbols matched within 90 s (range: 0–90, higher scores indicating better performance) [60] and the number of seconds required to complete the task (on average 29 and 75 s for parts A and B, with longer times suggesting higher impairment) [61]. The presence of cognitive decline or neurocognitive disorders was excluded as a result of a Mini-Mental State Examination (MMSE) total score > 26 [62].

The patient underwent laboratory exams and an electrocardiogram at baseline and a brain [^18^F]-fluorodeoxyglucose positron emission tomography integrated with computed tomography (^18^F-FDG PET/CT) after one week (i.e., almost one month after the beginning of 3-MeO-PCP consumption and ten days following the massive intake). A brain ^18^F-FDG PET/CT scan was performed only once as part of the initial assessment to support the description of clinical features. The exam was conducted with a Biograph mCT64 PET/CT scanner (Siemens Healthineers). The patient fasted for 6 h before the radiotracer injection. FDG PET/CT acquisition started 40 min after intravenous bolus injection of ^18^F-FDG (3.7 MBq/kg), while the subject rested quietly in a dimly lit and silent room. PET acquisition lasted 20 min, and images were reconstructed using an iterative time of flight algorithm with CT-based attenuation correction. After a visual examination, a semiquantitative analysis was performed through voxel-wise comparison using Statistical Parametric Mapping version 8 (SPM8, Wellcome Department of Cognitive Neurology, London, UK) to identify regional ^18^F-FDG-PET hypo- or hyper-metabolism. Patient images were compared with those of 12 male healthy controls (age range: 27–45 years), selected from a previously gathered database. The SPM8 normalization algorithm was employed to register ^18^F-FDG PET images with an MNI-based template of ^18^F-FDG, using the following settings: a 12-parameter affine transformation (3 for translations, 3 for rotations, 3 for shears, and 3 for zooms), a nonlinear frequency cut-off of 25 mm, 16 nonlinear iterations, and a nonlinear regularization switched at 1. A tri-linear interpolation was applied during the final re-slicing. Spatially normalized images were smoothed by convolution with an isotropic Gaussian kernel (full-width half maximum, FWHM = 8 mm) to increase the signal-to-noise ratio. Global normalization was performed using proportional scaling by setting the global cerebral blood flow value to 50 mL/dl/min. Statistical analysis was carried out through an unpaired 2-sample *t*-test with the SPM contrast set, respectively, at “−1,1” and “1,−1” to detect regional hypo- and hyper-metabolism compared to the control group. The resultant t-statistic was created with a threshold of *p* < 0.001 and a cluster extension of 120 voxels.

**Figure 1:**
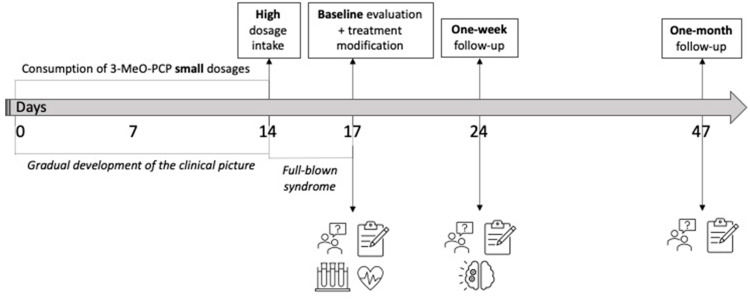
Case-history timeline.

A further clinical interview with psychometric and neuropsychological assessment was carried out at one-month follow-up, while ^18^F-FDG PET/CT was not repeated throughout the evaluation period. A timeline summarizing events and interventions has been provided in Figure 1.

After a thorough explanation of the procedures, the patient signed a written informed consent that included the use of de-identified clinical data for research purposes.

### 2.2. Results

The patient resulted in being ‘markedly ill’ according to baseline CGI-S (5) and BPRS (98, cut-off ≥53) total scores [63,64], as well as for the SCL90-R global severity index (2.61, cut-off >2). A total of 9 out of the 24 BPRS items were rated as ‘severe’ or ‘very severe’, specifically somatic concerns, elevated mood, grandiosity, suspiciousness, unusual thought content, bizarre behavior, self-neglect, conceptual disorganization, and tension. Accordingly, the patient displayed severe scores in every SCL90-R dimension, with the highest being ‘sleep disruptions’, ‘anxiety’, ‘phobic anxiety’, and ‘paranoid ideation’. The patient scored a total of 29 symbols out of 90 at the DSST (a little over a quarter of the possible correct answers), with one mistake during the performance, and took 30 s and 2 min to complete, respectively, TMT-A and B (with two mistakes in part B of the task). Laboratory tests showed heightened values of serum glutamic pyruvic transaminase/alanine-aminotransferase (54 UI/L) and an altered lipid profile (triglycerides 275 mg/dL, total cholesterol 265 mg/dL, low-density lipoproteins 166 mg/dL).

At the end of the first evaluation, the psychopharmacological treatment was modified as follows: paroxetine 10 mg/day, valproate 1 g/day, gabapentin 1.2 g/day, and olanzapine 10 mg/day. After one week of treatment, clinicians detected a 20% reduction in the BPRS total score, although the items ‘suspiciousness’, ‘unusual thought content’, and ‘conceptual disorganization’ were still rated as ‘severe’. Slightly lower SCL90-R scores were obtained by the patient for ‘sleep disruption’ and ‘anxiety’. The patient also showed a mild improvement in DSST (31/90 symbols), TMT-A (26 s), and TMT-B (1 min 58 s) scores, without mistakes during the performances.

**Figure 2:**
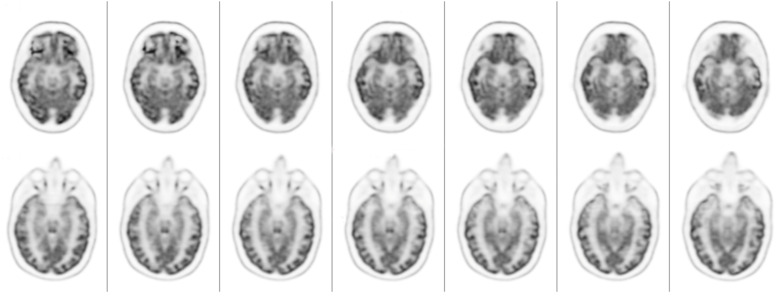
Brain 18F-FDG PET axial slices show a mild reduction in 18F-FDG uptake in the bilateral inferior frontal cortex and bilateral inferior temporal cortex.

Brain ^18^F-FDG PET/CT visual analysis showed a mild reduction in ^18^F-FDG uptake within the bilateral frontal inferior cortex and bilateral temporal inferior cortex (Figure 2).

**Figure 3:**
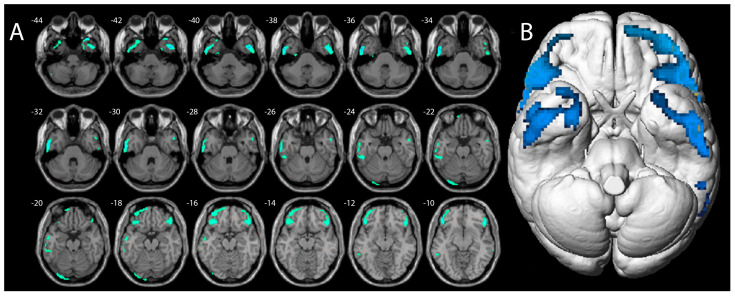
Axial magnetic resonance slices (**A**) and brain surface rendering (**B**) show the results of SPM analysis. The color-coded regions indicate the locations where the patients’ voxel values are significantly hypometabolic compared with the healthy control group (*p* < 0.001).

The voxel-wise analysis, using *p* values of <0.001, showed lower ^18^F-FDG-PET uptake in the bilateral inferior frontal cortex and bilateral temporal inferior cortex compared to healthy controls (Figure 3).

Specifically, clusters of major hypometabolic voxels were found in the right inferior frontal gyrus, with a peak at 58/30/4 mm (1036 voxels, T-Score 7.9), and in the inferior and middle temporal gyrus, with a peak at 46/2/−40 mm (522 voxels, T-Score 7.8). Conversely, by setting the voxel-wise analysis to reveal regions of increased ^18^F-FDG uptake, no voxels of significant hypermetabolism were found in comparison to healthy subjects (*p* values > 0.001).

**Figure 4:**
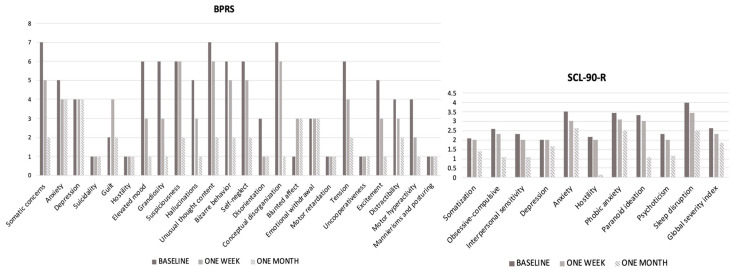
Psychometric assessment at different time points. *Abbreviations*: BPRS, Brief Psychiatric Rating Scale; SCL-90, Self-report Symptom Checklist Inventory 90—Revised version.

After one month of treatment, visual hallucinations, paranoid delusions, and thought disorganization, as well as psychomotor agitation, had totally disappeared, while some anxious and depressive symptoms were still reported by the patient (Figure 4). The neuropsychological performance improved as well based on DSST (42/90 symbols—almost half of the possible total score) and TMT Parts A and B (23 s and 1 min 21 s, respectively) scores (Table 2).

## 3. Discussion

To the best of our knowledge, this is the first report describing a substance-induced psychotic disorder following oral ingestion of 3-MeO-PCP, characterizing the clinical course over time, i.e., beyond the intoxication timeframe, and focusing on cognitive functioning with the support of a brain ^18^F-FDG PET/CT.

Following the repeated ingestion of small dosages of 3-MeO-PCP for almost a month and then a massive oral intake, the patient developed persistent psychotic symptoms and behavioral alterations and displayed poor performance on neuropsychological assessment. The impaired cognitive functioning was supported by a reduced uptake of ^18^F-FDG in some brain regions and was detectable one month after the beginning of 3-MeO-PCP consumption and about ten days after the consumption of a large amount.

### 3.1. Substance-Induced Psychosis: A Diagnostic Challenge

Substance-induced psychosis (SIP) has been generally depicted as a different variant of psychotic disorder, although the risk of progression to schizophrenia can be high and variable depending on the type of substance assumed [65]. The first mention of substances causing transient psychotic states that mimic positive and negative symptoms of schizophrenia dates to the 1960s, while the introduction of the term SIP with diagnostic criteria occurred in 1994 in the fourth edition of the DSM, remaining unchanged up to the latest versions of the manual [51]. A timeframe for the resolution of symptoms (from one to six months) is specified in both the DSM and the International Classification of Diseases [22,51,66]. However, diagnoses convert into primary psychotic disorders in up to 50% of cases, and growing evidence highlights a persistent syndrome following chronic use, which can endure over the identified timeframes and despite abstinence [65,67].

Differential diagnosis is challenging, considering estimates of 7–25% of misdiagnosis between first-episode psychosis and SIP. This raises the question of whether SIP could be considered a separate diagnostic entity, stable over time, and with distinct clinical and psychopathological features only partially recognized by the current classification systems [67]. Visual and kinesthetic hallucinations, persecutory and paranoid delusions (highly related to the abnormal perceptions and based on processes of confirmation and interpretation), depersonalization and derealization associated with affective symptoms, disorganized behaviors, motor hyperactivity (often resulting in aggressiveness), and sufficient (albeit fluctuating) levels of insight have been reported among the main characteristics of SIP [52,67,68,69].

Compared to schizophrenic patients, it has been noted that subjects with SIP display a higher age-at-onset and less frequent family history of psychosis, with higher rates of relatives with substance use [68,69]. The prognosis can vary according to the type of substance consumed, and young adults are considered most at risk of progression to a severe mental illness within two years from the onset of SIP [68]. Independently of the exact diagnosis or the pathophysiology, the comorbidity between substance use and severe mental illness is burdened by negative outcomes, including non-adherence, increased relapse, and more frequent hospitalizations [69], suggesting the need for timely and more effective therapeutic interventions.

### 3.2. NPS-Induced Psychosis: Focus on 3-MeO-PCP

It is noteworthy that the occurrence of drug-induced psychosis, besides the severity of use, is highly related to the pathogenetic mechanism (e.g., serotonergic, dopaminergic, glutamatergic) related to the pharmacodynamics of substances [52]. Although a primary mechanism of action can be generally identified, the frequent use of multiple substances and the fact that NPS exert their effects on several pathways can determine an overlap of symptoms and neurobiological underpinnings [67].

Since PCP animal models of psychosis were first proposed about 20 years ago, glutamatergic hypotheses have been increasingly accepted among the etiopathological underpinnings of schizophrenia and are primarily based on glutamate NMDA receptor antagonism [14]. However, there is evidence of actions other than NMDA and mGlu2/3 metabotropic receptor antagonism in the prefrontal cortex that might underlie psychotic symptoms [52,67].

As for the clinical consequences of substances primarily acting on glutamatergic pathways (e.g., 3-MeO-PCP and other PCP-like compounds), dissociation, bodily and kinesthetic hallucinations, near-death experiences, and detachment from the environment, along with somatic delusions, negative affective symptoms, irritability and, sometimes, aggressiveness, seem to be prominent [49,50].

Clinical features induced by 3-MeO-PCP described in this patient are in line with previous findings and are potentially sustained by the neural correlates highlighted at the brain ^18^F-FDG PET/CT. According to the disease model of addiction, SUD relies on the interplay of biological, psychological, and social factors, which can lead to long-lasting neuroadaptations in brain structure and functioning (e.g., in neurotransmission and blood flow) [70]. The application of neuroimaging techniques to the field has contributed to a better understanding of the impact of psychotropic substances both at the anatomical and physiological levels, highlighting the involvement of several regions and networks that are in charge of executive function, memory, reward, and stress response [71].

The effects of 3-MeO-PCP could be explained within previous evidence reporting the role of glutamatergic dysfunctions in corticostriatal networks in the development of addiction [72], as well as the relationship between drug-induced psychotic symptoms and alterations in multiple brain regions following the acute administration of NMDA receptor antagonists [73]. As for the deficits in specific brain areas identified at the brain scan, it should be noted that chronic use of PCP-like compounds is associated with a reduced gray matter volume in the bilateral frontal cortex [53] and, more recently, the involvement of additional brain regions, including the inferior temporal cortex, has been recognized as a distinct feature of subjects with SUD [71]. Regarding behavioral abnormalities, recent studies highlighted a long-lasting activation of the medial prefrontal cortex only after a systemic administration of PCP, suggesting that the induced locomotor activity and behavioral stereotypes might be mediated by inputs from regions outside the prefrontal cortex [74]. Therefore, a composite network should be more properly argued for the complex clinical presentation.

Besides the psychotic and behavioral manifestations that are frequently observed in SIP, little is known about the cognitive profile, and mixed results have been collected so far [52]. A recent systematic review reported that, similarly to schizophrenic patients, cognitive impairment is frequent in SIP with distinct deficits in attention, working memory, processing speed, executive functioning, social cognition, and worse global functioning than non-psychotic SUD patients, and outlined unique differences for the visual-based processing [65].

### 3.3. The Effects of 3-MeO-PCP on Cognitive Functioning and Brain Metabolism

Like other PCP-like compounds, some cognitive changes have been described among the acute and chronic consequences of 3-MeO-PCP ingestion [50]. Cognitive deficits, i.e., selectively reduced memory capacity, have been reported for two other NMDA receptor antagonists, PCP and methoxetamine, in animal models [75]. Further, alterations of neuropsychological tasks and an impaired brain metabolism in prefrontal cortex areas, as revealed by ^18^F-FDG PET/CT images, were reported in the case of a 23-year-old man with methoxetamine-induced psychotic disorder [53]. Unlike dopamine, NMDA receptors are widely distributed throughout the brain, including sensory and association cortices and subcortical areas, and it has been hypothesized that such a distribution pattern might affect higher-order cognitive dysfunctions as detected by behavioral, neurophysiological, and functional imaging studies in schizophrenia [13].

Here, poor neuropsychological performance was observed at baseline and one-week follow-up both during DSST and TMT execution, which cannot be attributed to any previous cognitive impairment of the patient given his young age, the absence of neurological diseases in his medical history, or the MMSE score > 26.

Scores on the DSST seem quite in line with average values (±standard deviations) reported by previous studies on psychiatric patients with different diagnoses, e.g., with depression (32.4 ± 15.8) and schizophrenia (38.18 ± 10.47), and remarkably compromised if compared to healthy controls (62.31 ± 11.92) [76,77]. Performance on the DSST represents the final common pathway for the expression of different types of impairment, and the integrity of several mental functions is required to perform well on the task, such as motor speed, attention, visual perception, associative learning, and working memory [57]. Moreover, the role of executive functioning has been demonstrated to contribute to the performance of DSST and to correlate with the activation of specific cerebral areas, such as the frontal lobes [78].

The execution of TMT involves several cognitive functions as well and depends on both prefrontal and non-frontal brain region activity, although there are some differences between the two parts of the task [79,80]. Indeed, it has been reported that TMT-A scores can be considered a measure of attention and speed that mainly relies on visual scanning and graphomotor and visuomotor processing speed, while working memory, inhibition control, or set-switching abilities seem to be more specifically implicated in TMT-B performance [79].

Executive functioning should be considered a multifaceted construct comprising several higher-order cognitive processes that ensure flexibility, self-regulation, strategic planning, and goal-directed actions [81]. Although the physiological substrates have been mostly ascribed to the frontal lobes, the involvement of a more widespread network, which extends beyond the frontal cortex and encompasses additional brain areas, has been argued [82]. A thorough, reciprocal interconnection between the nodes of the fronto-parietal network and with other association cortices and subcortical areas is necessary to access several high-level cognitive processes, and dysfunctions in some of these regions can be differentially detected by neuropsychological tests [81].

A previous analysis of resting brain metabolism and neuropsychological performance in schizophrenic patients showed similar cognitive deficits and highlighted that hypometabolism in the frontal lobes (i.e., superior, middle, and inferior frontal gyruses bilaterally) may be the substrate for a deficient performance on some tasks, specifically TMT part B [83]. Conversely, both animal and human studies on long-term exposure to PCP reveal metabolic hypofunction and deficits in the frontal lobes, which mimic the hypofrontality typically detected in schizophrenia [84].

Within this framework, it has been reported that distinct networks can underlie different cognitive abnormalities in SUD, and a differential association has been found between specific executive functions (such as inhibition and flexibility on one side, self-regulation, decision-making, and emotion processing on the other) and the regional metabolism in the prefrontal cortex, frontal gyrus, and temporoparietal regions [85]. Further, reduced impulse control has been noticed in relation to decreased metabolic rates in the frontal executive areas, as well as in alcoholic samples [86].

Thus, it could be hypothesized that 3-MeO-PCP consumption led to impaired patient performance on neuropsychological tasks (especially on baseline part B of TMT, with two mistakes) and that it might relate to the hypometabolism in bilateral frontal and temporal cortices detected at brain ^18^F-FDG PET/CT. As reported in this case history, a comprehensive assessment (combining laboratory exams, psychometric and neuropsychological tests, and functional neuroimaging techniques) might better support the investigation of complex clinical features and their underlying mechanisms, possibly unveiling new targets for prevention and intervention strategies, which are particularly needed in a constantly changing field like that of NPS. It has been recently shown that combining brain-wide positron emission tomography, transcriptomic data (e.g., receptor distribution), and computational neuroimaging methods can reproduce the effect of specific substances on the human brain [87]. Indeed, the integration of functional and molecular-level alterations might help identify markers of interindividual variability and pave the way for precision psychiatry approaches.

### 3.4. Limitations

There are some limitations that must be acknowledged and suggest caution in interpreting these findings, such as the lack of information about the exact blood concentration of 3-MeO-PCP, the concomitant assumption of drugs during the execution of ^18^F-FDG PET/CT, and the presence of previous multiple substance use in the patient’s history, which might prevent us from unambiguously attributing brain image findings to 3-MeO-PCP effects, as well as the missing longitudinal neuroimaging follow-up. Moreover, a specific NPS may cause different psychopathological effects according to the individual vulnerability [49]. High levels of comorbidity in subjects using substances and developing SIP can usually be detected [67], and the self-medication theory has been discussed for several NPS uses, including 3-MeO-PCP, mainly to overcome symptoms of ADHD, anxiety, and depression [10]. Indeed, also the patient from this case had reported the presence of anxious-depressive symptoms during adolescence and youth. Thus, present results might be poorly generalizable to wider and more representative samples.

However, considering the absence of a SUD diagnosis and the relatively recent detoxification program, the displayed symptomatology and the brain ^18^F-FDG PET/CT findings could be quite reliably ascribed to the compound. Therefore, this is the first report describing repeated oral ingestion of 3-MeO-PCP with subsequent occurrence and persistence of psychotic symptoms and highlighting the possibility of brain impairment, detectable both through neuropsychological tests and neuroimaging, in young, healthy subjects such as the 29-year-old man in this case. Therefore, the additional contribution to the field may be suggested by considering that, compared to previous reports of acute intoxication, in our study, the clinical picture was observed over a longer period, a multidimensional assessment was performed, and particular attention was paid to cognitive effects. Although cognitive impairment following acute and chronic intake of PCP-analogues has been mentioned in the literature, the administration of specific neuropsychological tasks and the ^18^F-FDG scan allowed us to describe the cognitive functions affected and the neurofunctional substrates involved more precisely.

## 4. Conclusions

The spread of NPS has been rapidly and progressively modifying the worldwide drug scene. The high rates of consumption among vulnerable populations, like adolescents, young adults, and psychiatric patients, the wide range of medical and psychopathological consequences, as well as the frequent acute intoxications and deaths, make NPS a challenge for public health [49]. The peculiar clinical features and the variety of neurobiological pathways involved require clinicians to constantly update not only about the range of NPS and their psychoactive effects but also about their toxicity and safety profile [50].

Further research about clinical phenomenology and underlying mechanisms is needed to identify better therapeutic approaches to manage both acute and long-term consequences of NPS intake, including the occurrence of substance-induced psychotic disorders [67]. In this view, our findings outline the need for carefully warning clinicians, psychiatric patients, and the general population about the risks of NPS use in relation to cognitive functioning.

More neuroimaging studies on NPS with longitudinal follow-up are certainly needed to assess whether their effects are limited to recreational consumption or imply more long-term consequences, especially on cognition, and if the latter should be considered independent from the development of psychotic symptoms/disorders. Although domain-specific dysfunctions could not be identified, phenotyping the cognitive profile of SIP might help tailor the therapeutic approach and, besides allowing a more precise differentiation from other psychotic disorders, provide targets for specific treatment strategies, such as cognitive remediation techniques that showed some effectiveness as adjunctive treatments in both SUD and schizophrenia [88,89]. Multidomain functional neuroimaging has been shown to be predictive of illness progression and outcomes in both neurological and psychiatric diseases [90,91], including SUD [92]. In this light, a multidisciplinary assessment (including more informative techniques like PET/CT scans) could provide clinicians with more accurate diagnostic tools and help develop more personalized prevention strategies and treatments based on a better understanding of neuroanatomy and neurobiology implied in substance use [71].

## Figures and Tables

**Table 1:** Intoxication cases with 3-MeO-PCP from the literature. *Abbreviations*: 3-MeO-PCP: 3-Methoxyphencyclidine; F: female; M: male; UK: United Kingdom; USA: United States of America.

Authors	Country	Number of Cases	Gender, Age (Years)	Biological Samples	Other Substances/Drugs	Outcome	3-MeO-PCP Concentration (ng/mL)
Allard et al., 2019 [26]	France	1	M, 17	Whole blood, Urine	No	Nonfatal	Not quantified
Ameline et al., 2019 [33]	France	2	M, 41	Femoral blood, Urine	Yes	Fatal	Blood: 498, Urine: 16,700
			F, 39	Femoral blood, Urine, Bile	Yes	Fatal	Blood:63, Urine: 94, Bile: 64
Ameline et al., 2019 [34]	France	1	M, 41	Femoral blood, Urine	Yes	Fatal	Blood: 498, Urine: 16,700
Arbouche et al., 2021 [35]	France	1	M, 44	Femoral blood, Urine	Yes	Fatal	Blood: 3525, Urine: 7384
Backberg et al., 2015 [29]	Sweden	59 (recorded from STRIDA Project)	M and F, 14–55	Serum, Urine	Yes	Nonfatal	Serum range: 1–242 Urine range: 2–52,759
Bakota et al., 2016 [36]	USA	1	M, 29	Femoral blood, Heart blood	Yes	Fatal	Blood: 139
Berar et al., 2019 [30]	France	1	M, 17	Whole blood, Urine	No	Nonfatal	Blood: 71, Urine: 701
Bertol et al., 2017 [8]	Italy	2	M, 19	Whole blood, Urine	Yes	Nonfatal	Blood: 350, Urine: 6109
			M, 21	Whole blood, Urine	Yes	Nonfatal	Blood: 180, Urine: 3003
Chang et al., 2017 [37]	USA	1	M, 27	Urine	No	Nonfatal	Not quantified
Copeland et al., 2022 [25]	UK	1	M, 33	Femoral blood, Urine	Yes	Fatal	Blood: 48 Urine: 196
de Jong et al., 2019 [38]	The Netherlands	1	M, 30	Serum, Femoral blood	Yes	Fatal	Serum: 123, Femoral blood: 152
Frison et al., 2021 [32]	Italy	2	-	Serum, Whole blood, Urine	No	Nonfatal	Not quantified
Gomila et al., 2019 [39]	Spain	2	F, 32	Serum, Urine	Yes	Nonfatal	Serum: 47, Urine: 9645
			M, 24	Urine	Yes	Nonfatal	Urine: 561
Grossenbacher et al., 2019 [40]	France	5	M, 36	Blood, Urine	No	Nonfatal	Not quantified
			M, 32	Urine	No	Nonfatal	Not quantified
			-	Urine	No	Nonfatal	Not quantified
			M, 41	Femoral blood, Urine	Yes	Fatal	Blood: 498, Urine: 16,700
			F, 39	Femoral blood, Urine	Yes	Fatal	Blood: 63, Urine: 94
Kintz et al., 2019 [42]	France	1	F, 39	Femoral blood, Urine, Bile, Hair	Yes	Fatal	Blood: 63, Urine: 94, Bile: 64
Helander et al., 2015 [41]	Sweden	4 (recorded from STRIDA Project)	M and F, 27–48	Serum, Urine	Yes	Nonfatal	Not quantified
Johansson et al., 2017 [27]	Sweden	8	M, 27	Femoral blood	No	Fatal	380
			M, 21	Femoral blood	Yes	Fatal	180
			M, 27	Femoral blood	Yes	Fatal	230
			M, 29	Femoral blood	Yes	Fatal	120
			M, 32	Femoral blood	Yes	Fatal	60
			M, 27	Femoral blood	Yes	Fatal	50
			F, 20	Femoral blood	Yes	Fatal	80
			M, 19	Whole blood	No	Nonfatal	140
Krotulski et al., 2018 [43]	USA	1	M, 31	Peripheral blood, Urine	Yes	Fatal	Blood: 1, Urine: 32
Leciñena et al., 2019 [44]	Spain	2	F, 32	Urine	Yes	Nonfatal	Not quantified
			M, 24	Serum, Urine	Yes	Nonfatal	Serum: 47
Mitchell-Mata et al., 2017 [45]	USA	2	M, 21	Peripheral blood	Yes	Fatal	Blood: 3200
			M, 58	Central blood, Urine	Yes	Fatal	Blood: 630
Pelletier et al., 2021 [3]	France	1	M, 37	Peripheral blood, Urine	Yes	Nonfatal	Urine: 1,100,000
Stevenson et al., 2014 [46]	UK	1	M, 20	-	Yes	Nonfatal	-
Thornton et al., 2017 [47]	USA	1	M, 27	Whole blood, Urine	Yes	Nonfatal	Serum: 167
van den Bersselaar et al., 2021 [48]	The Netherlands	1	M, 40	-	No	Nonfatal	-
Zidkova et al., 2017 [31]	Czech Republic	2	M, 37	Whole blood, Urine	Yes	Nonfatal	Serum: 49
			M, 40	Whole blood, Urine	Yes	Nonfatal	Serum: 66

**Table 2:** Neuropsychological assessment at different time points. *Abbreviations*: DSST: Digit Symbol Substitution Test; TMT: Trail Making Test.

	Baseline	One Week	One Month
DSST ^a^	29	31	42
TMT-A ^b^	30	26	23
TMT-B ^b^	120	118	81

^a^ number of correct symbols within 90 s; ^b^ seconds.

## Data Availability

No new data were created or analyzed in this study. Data sharing is not applicable to this article.
